# The Effect of Peripheral Nerve Block on Postoperative Delirium in Older Adults Undergoing Hip Surgery: A Systematic Review and Meta-Analysis of Randomized Controlled Trials

**DOI:** 10.3390/jcm12072459

**Published:** 2023-03-23

**Authors:** Su Yeon Kim, Ha Young Jo, Hyo-Seok Na, Sung-Hee Han, Sang-Hwan Do, Hyun-Jung Shin

**Affiliations:** Department of Anesthesiology and Pain Medicine, Seoul National University Bundang Hospital, Seongnam 13620, Republic of Korea

**Keywords:** elderly, hip surgery, meta-analysis, peripheral nerve blocks, postoperative delirium

## Abstract

This meta-analysis aimed to determine whether peripheral nerve blocks (PNB) reduce postoperative delirium (POD) in elderly patients undergoing hip surgery. This study was registered in the International Prospective Register of Systematic Reviews (PROSPERO; CRD42022328320). The PubMed, EMBASE, Web of Science, and Cochrane Library databases were searched for randomized controlled trials (RCTs) on 26 April 2022. A total of 19 RCTs with 1977 participants were included. Perioperative PNB lowered the POD incidence on the third postoperative day (OR: 0.59, 95% CI [0.40 to 0.87], *p* = 0.007, *I*^2^ = 35%), in patients without underlying cognitive impairment (OR: 0.47, 95% CI [0.30 to 0.74], *p* = 0.001, *I*^2^ = 30%), and when a fascia iliaca compartment block (OR: 0.58, 95% CI [0.37 to 0.91], *p* = 0.02, *I*^2^ = 0%) or a femoral nerve block (OR: 0.33, 95% CI [0.11 to 0.99], *p* = 0.05, *I*^2^ = 66%) were performed. The pain score was also reduced (SMD: −0.83, 95% CI [−1.36 to −0.30], *p* = 0.002, *I*^2^ = 95%) after PNB. Perioperative PNB can lower the POD incidence and pain scores up to the third postoperative day. However, considering the wide variety of PNBs performed, more trials are needed to identify the effects of each PNB on POD.

## 1. Introduction

The Diagnostic and Statistical Manual of Mental Disorders, Fifth Edition of the American Psychiatric Association, describes delirium as a disturbance in attention or cognition [1]. Delirium can occur newly, or is exacerbated in patients with pre-existing cognitive impairment, after surgery [2]. The incidence of postoperative delirium (POD) varies from 11–40%, depending on the surgery type [3,4]. POD increases the length of hospital or intensive care unit stay, the likelihood of reoperation or readmission to the intensive care unit, medical costs, and in-hospital mortality [5]. POD can last from 10 min after anesthesia to 7 days or until discharge [6]. Delirium can be classified into hyperactive, hypoactive, and mixed forms, according to motor activity [7]. There are several tools for diagnosing delirium, including the Confusion Assessment Method (CAM) or the Richmond Agitation-Sedation Scale (RASS). The Memorial Delirium Assessment Scale (MDAS) and the Delirium Rating Scale-Revised-98 (DRS-R98) are also useful for assessing the severity of delirium [7].

Many factors are known to cause POD, including old age, underlying diseases such as diabetes mellitus and hypertension, prior cognitive impairment, drinking history, drug abuse, the perioperative lactic acid level, low albumin, intra-operative blood transfusion, fluid and electrolyte imbalances, and anemia [8,9,10,11]. Pain is thought to be a major risk factor for POD based on research showing that patients with inadequately controlled pain are more likely to develop delirium [12]. Therefore, sufficient pain relief may contribute to a reduction in the incidence of POD. In terms of enhanced recovery after surgery (ERAS), a nerve block is frequently performed as a method of reducing pain [13].

Hip surgery is a high-risk procedure with a POD incidence of up to 17%, and this risk increases in older patients [14]. Currently, for patients with hip fractures, various nerve blocks, such as the psoas compartment block, fascia iliaca block, femoral nerve block, lateral femoral cutaneous nerve block, or obturator nerve block, are used for pain control. Among these, the fascia iliaca compartment and femoral nerve blocks are effective in reducing postoperative pain [15]. Since pain is one of the strongest risk factors for POD, a peripheral nerve block (PNB) may contribute to lowering the occurrence of POD by providing effective analgesia after surgery. Based on this hypothesis, clinical studies have shown conflicting results [16,17,18,19].

This study aimed to investigate whether PNB reduces the incidence of POD in older adults undergoing hip fracture surgery, according to the type of PNB and underlying cognitive disorder, in particular.

## 2. Materials and Methods

### 2.1. Study Design

This study was conducted according to the Preferred Reporting Items for Systematic Reviews and Meta-Analysis (PRISMA) statement [20] and registered in the International Prospective Register of Systematic Reviews (PROSPERO; identifier: CRD42022328320). Ethical review and approval were waived for this study because data from published resources were used for analyses.

### 2.2. Information Sources and Literature Search

We searched PubMed, EMBASE, Web of Science, and Cochrane Library databases on April 26 2022. The search terms included those related to hip fracture, peripheral nerve block, and delirium, such as “intertrochanter”, “trochanter”, “surgery”, “nerve block”, and “cognitive impairment”, along with Medical Subject Heading or EMBASE Subject Heading terms. The search strategy used in this study is described in Supplementary File S1. There were no restrictions on the year of publication or language.

### 2.3. Study Selection

After searching the databases mentioned above, two authors (S.K. and H.S.) selected the final studies by independently reviewing the titles, abstracts, and full texts of the remaining articles in sequence. In cases of disagreement, a decision was made through discussion.

The inclusion criteria were as follows: (1) randomized controlled trials, (2) patients who underwent hip fracture surgery, (3) cases of peripheral nerve block performed perioperatively, (4) evaluation of the incidence of POD, and (5) literature with a control group.

The exclusion criteria were as follows: (1) case reports, (2) observational studies, (3) retrospective cohort studies, (4) review articles, (5) trial protocols, (6) editorials, and (7) animal studies. We did not exclude gray literature to reduce the possibility of publication bias [21].

### 2.4. Data Extraction

We reviewed the articles and extracted the following data: age, the total number of participants, study design, type of surgery, type of nerve block, the local anesthetic used, POD incidence, and postoperative pain scores. When data were presented in graphs, they were extracted using the online tool WebPlotDigitizer (version 4.6; WebPlotDigitizer, A. Rohatgi, Pacifica, CA, USA). If the data were described as median (interquartile range), we estimated the mean and standard deviation using the equation presented by Wan et al. [22]. To determine the frequency of POD occurrence, when the cognitive state was described by continuous variables, we contacted the authors [23,24,25], requesting data on the number of patients with POD occurrence; however, none of them replied.

### 2.5. Assessment of Risk of Bias

Two authors (S.K. and H.S.) independently evaluated the quality of the articles and discussed them to reach an agreement in case of discordance. The risk of bias was assessed in six domains: randomization process, deviations from intended interventions, missing outcome data, measurement of the outcome, selection of the reported result, and overall bias covering the above categories. The risk of bias of each article was graded as “low”, “some concerns”, or “high”, using the Risk of Bias 2 tool supplied by the Cochrane Collaboration [26].

### 2.6. Grading the Quality of Evidence

The quality of evidence for each outcome was assessed based on the grading of recommendations, assessment, development, and evaluation (GRADE) [27]. The quality of the evidence was assessed as very low, low, moderate, or high. GRADE was assessed using GRADEpro (McMaster University, Hamilton, ON, USA, 2021) (Supplementary File S2).

### 2.7. Outcome Measures

The primary outcome was the incidence of POD on postoperative days three and seven. The secondary outcome was the postoperative pain scores measured at postoperative days one through three during the resting state. When pain scores were measured at multiple time points, the closest value to 72 h postoperatively was recorded.

### 2.8. Statistical Analysis

We calculated the standardized mean differences (SMDs) and 95% confidence intervals (CI) for continuous outcomes. Odds ratios (OR) and the 95% CI were calculated for the dichotomous data. We performed the analyses with the random-effects models using inverse variance for both continuous and dichotomous outcome analyses due to different effect sizes and interventions across the included studies. A sensitivity analysis using the leave-one-out method was performed for each meta-analysis. Higgins’ *I*^2^, the heterogeneity statistic Cochrane’s Q, and the corresponding *p*-values were calculated for the heterogeneity tests. Heterogeneity was considered high when *I*^2^ was >50%. A funnel plot was presented with OR against the associated SEs to evaluate publication bias. Publication bias was considered if *p*-value < 0.1, using Egger’s linear regression test.

Review Manager (RevMan, version 5.4.1, the Cochrane Collaboration) and R software (version 4.1.3, R Foundation for Statistical Computing, Austria) were used for all analyses.

## 3. Result

### 3.1. Study Selection and Characteristics

**Figure 1:**
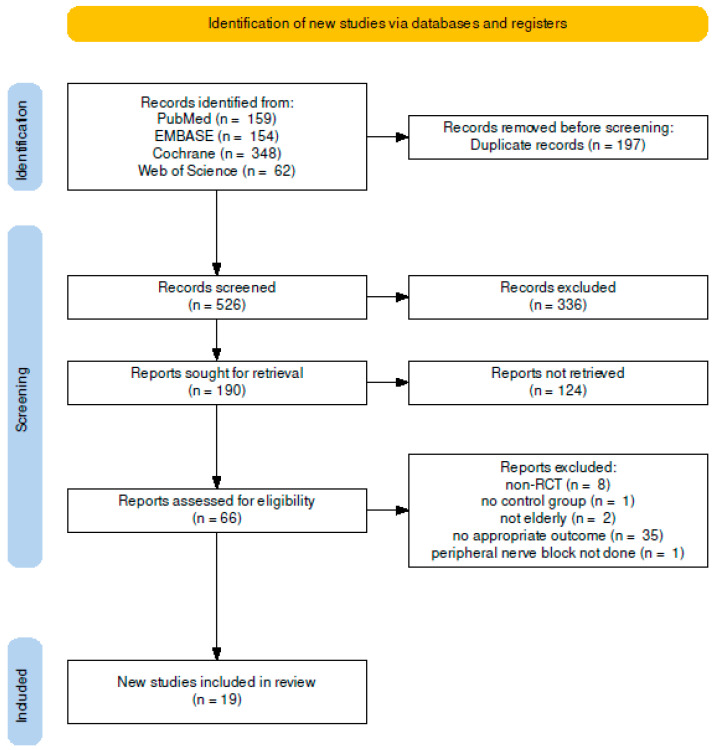
Flow diagram of study selection.

Two authors extracted 723 articles after an initial search from the PubMed (n = 159), EMBASE (n = 154), Cochrane Library (n = 348), and Web of Science (n = 62) databases, and 197 duplicate articles were removed. Two authors screened the remaining articles independently and excluded 336 and 124 articles based on the title and abstract, respectively. A full-text review was conducted of the remaining 66 articles, and 47 articles were excluded. The specific reasons for excluding each article are shown in Figure 1.

The characteristics of the 19 included studies are presented in Table 1. A fascia iliaca compartment block and femoral nerve block were performed in seven studies [16,17,18,19,28,29,30] and five studies [31,32,33,34,35], respectively. The following types of nerve blocks were performed in the remaining studies: lumbosacral plexus block [36,37], lumbar plexus block [38], a combination of psoas compartment block and sciatic nerve block [39], femoral and lateral cutaneous block [40], a combination of fascia iliaca compartment block and sciatic nerve block [41], and a combination of fascia iliaca compartment block, sacral plexus block, and superior cluneal nerve block [42]. In six studies, the local anesthetic was continuously administered through a catheter [17,19,31,32,38,39]. The details of the demographics and interventions for each study are described in Table 1.

### 3.2. Risk of Bias Assessment

**Figure 2:**
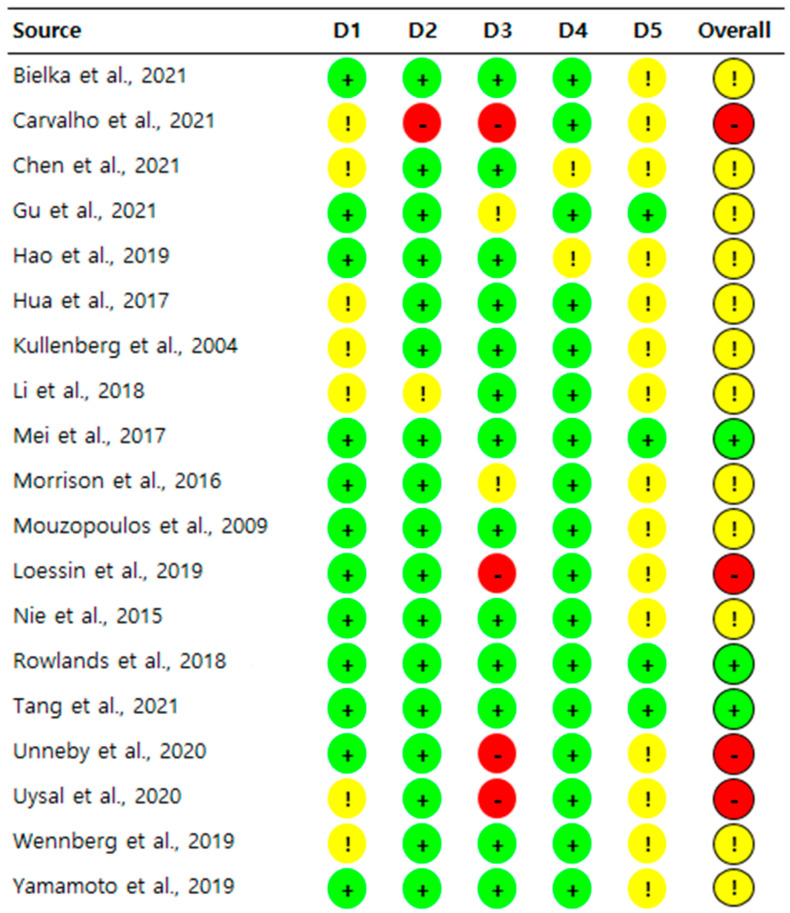
Risk of bias summary [16,17,18,19,28,29,30,31,32,33,34,35,36,37,38,39,40,41,42]. D1, randomization process; D2, deviations from the intended interventions; D3, missing outcome data; D4, measurement of the outcome; D5, selection of the reported result; 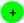, low risk; 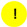, some concerns; 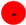, high risk.

Three studies [32,36,37] were classified as “low risk”, twelve studies [16,18,19,28,29,30,31,35,38,39,41,42] as “some concerns”, and four studies [17,33,34,40] as “high risk” in the overall bias (Figure 2). For bias arising from the randomization process, 12 studies [17,18,19,28,30,31,32,33,36,37,39,42] were rated as “low risk”, while 7 studies [16,29,34,35,38,40,41] were classified as “some concerns.” For bias due to deviations from the intended interventions, all but one study [40] with “high risk” due to lack of information, and another study [38], with “some concerns”, were assessed as “low risk.” For the bias caused by missing outcome data, two studies [31,42] were classified as “some concerns”, and four studies [17,33,34,40] were rated as “high risk” for the following reasons: insufficient information [17,40], and large population of exclusion after enrollment [33,34]. Two studies [28,41] were assessed as “some concerns”, while all other studies were assessed as “low risk” for bias in the measurement of the outcome. Meanwhile, all but four studies [32,36,37,42] evaluated as “low risk” were assessed as “some concerns” for bias in the selection of the reported result.

### 3.3. Meta-Analysis

In a study by Bielka et al. [39], three groups were included: (1) PNB group, (2) no-block group under spinal anesthesia, and (3) no-block group under general anesthesia. Among the groups, the comparison between the nerve block and no block groups under spinal anesthesia was included in the present data synthesis. One study [33] was only included in the meta-analysis of POD on postoperative day seven, not on postoperative day three, because this study presented POD incidence only on postoperative day seven. In another study [37], POD incidence was measured primarily on postoperative day seven; however, in case of early discharge prior to postoperative day seven, POD was measured at the time of discharge. Therefore, this study was excluded from the analysis of POD incidence on postoperative day three, but was only included in the analysis on postoperative day seven. One study [38] measured POD on postoperative day one, three, and seven; therefore, we used data from postoperative days three and seven for the analysis. Seven studies [16,18,19,31,32,39,41] did not describe the timing of POD assessment, while two studies [29,30] investigated POD one day after surgery. We categorized these randomized controlled trials (RCTs) as eligible for the analysis of POD three days after surgery.

Two tools were used to assess pain scores in the included studies: the visual analog scale (VAS) [16,18,28,30,33,34,36,37,38] and the numerical rating scale (NRS) [19,31,32,39]. We calculated the SMDs due to the conversion of data with median and interquartile ranges to mean and standard deviations.

#### 3.3.1. Postoperative Delirium at Postoperative Day Three

**Figure 3:**
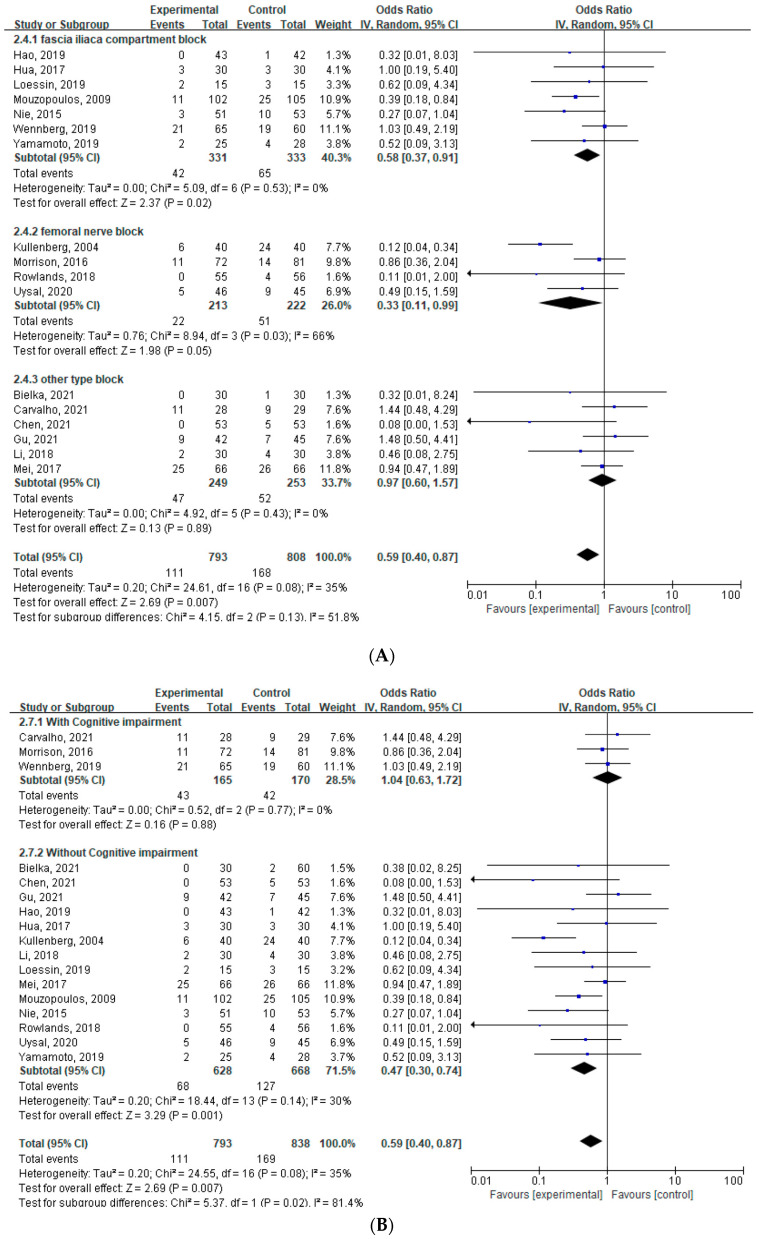
Forest plot: the effect of a peripheral nerve block on postoperative delirium at postoperative day three [16,17,18,19,28,29,30,31,32,34,35,36,38,39,40,41,42]. (**A**) Forest plot with subgroup analysis regarding the type of block. (**B**) Forest plot with subgroup analysis regarding underlying cognitive impairment. IV: inverse variance.

A total of 17 studies [16,17,18,19,28,29,30,31,32,34,35,36,38,39,40,41,42] (n = 1631; 793 in the PNB group and 838 in the control group) were included in this meta-analysis. The incidence of POD was lower in the PNB group than in the control group on postoperative day three (OR: 0.59, 95% CI [0.40 to 0.87], *p* = 0.007, *I*^2^ = 35%) (Figure 3). In a subgroup analysis regarding the type of nerve block, both the fascia iliaca compartment block (OR: 0.58, 95% CI [0.37 to 0.91], *p* = 0.02, *I*^2^ = 0%) and the femoral nerve block (OR: 0.33, 95% CI [0.11 to 0.99], *p* = 0.05, *I*^2^ = 66%) lowered the occurrence of POD, while other types of blocks or combinations of blocks did not show any significant effect on the development of POD compared with no block (Figure 3). In the subgroup analysis according to pre-existing cognitive impairment, the incidence of POD was lower when PNB was performed in patients without pre-existing cognitive impairment (OR: 0.47, 95% CI [0.30 to 0.74], *p* = 0.001, *I*^2^ = 30%) (Figure 3). In contrast, PNB did not affect the occurrence of POD in patients with pre-existing cognitive impairment (OR: 1.04, 95% CI [0.63 to 1.72], *p* = 0.88, *I*^2^ = 0%) (Figure 3).

In the sensitivity analysis, the effect size of delirium measured at postoperative day three decreased (OR: 0.72, 95% CI [0.53 to 0.96], *p* = 0.03, *I*^2^ = 0%) compared to the pooled effect after excluding one study, which was an outlier [35]. 

Most of the included studies were rated as having “some concerns” regarding the risk of bias assessment.

#### 3.3.2. Postoperative Delirium at Postoperative Day Seven

**Figure 4:**
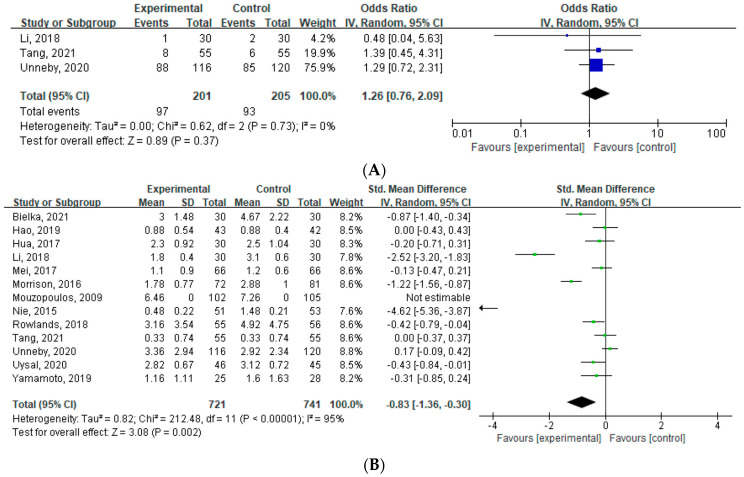
Forest plot [16,18,19,28,30,31,32,33,34,36,37,38,39]. (**A**) Effect of a peripheral nerve block on postoperative delirium at postoperative day seven. (**B**) Effect of a peripheral nerve block on postoperative pain. IV: inverse variance.

A meta-analysis of three studies [33,37,38] (n = 406; 201 in the PNB group and 205 in the control group) showed no significant differences in POD incidence between the two groups (OR: 1.26, 95% CI [0.76 to 2.09], *p* = 0.37, *I*^2^ = 0%) (Figure 4). 

In the sensitivity analysis, there was no significant difference in POD incidence (OR: 1.16, 95% CI [0.41 to 3.23], *p* = 0.78, *I*^2^ = 0%) after excluding an outlier [33].

Regarding the risk of bias assessment, one study [37] was rated as “low”, one [38] as “some concerns”, and the other [33] as “high.”

#### 3.3.3. Postoperative Pain Score

A meta-analysis of 13 studies [16,18,19,28,30,31,32,33,34,36,37,38,39] (n = 1462; 721 in the PNB group and 741 in the control group) showed a significant reduction in the postoperative pain score (SMD: −0.83, 95% CI [−1.36, −0.30], *p* = 0.002, *I*^2^ = 95%) (Figure 4).

In the sensitivity analysis, performing a peripheral nerve block was significantly more effective in reducing pain (SMD: −0.50, 95% CI [−0.87, −0.14], *p* = 0.007, *I*^2^ = 89%) after excluding an outlier [19]. However, this interpretation requires caution because the heterogeneity remains high.

Regarding the risk of bias assessment, most included studies [16,18,19,28,30,31,38,39] were rated as “some concerns”, while three studies [32,36,37] were rated as “low”, and two studies [33,34] as “high.”

#### 3.3.4. Publication Bias

**Figure 5:**
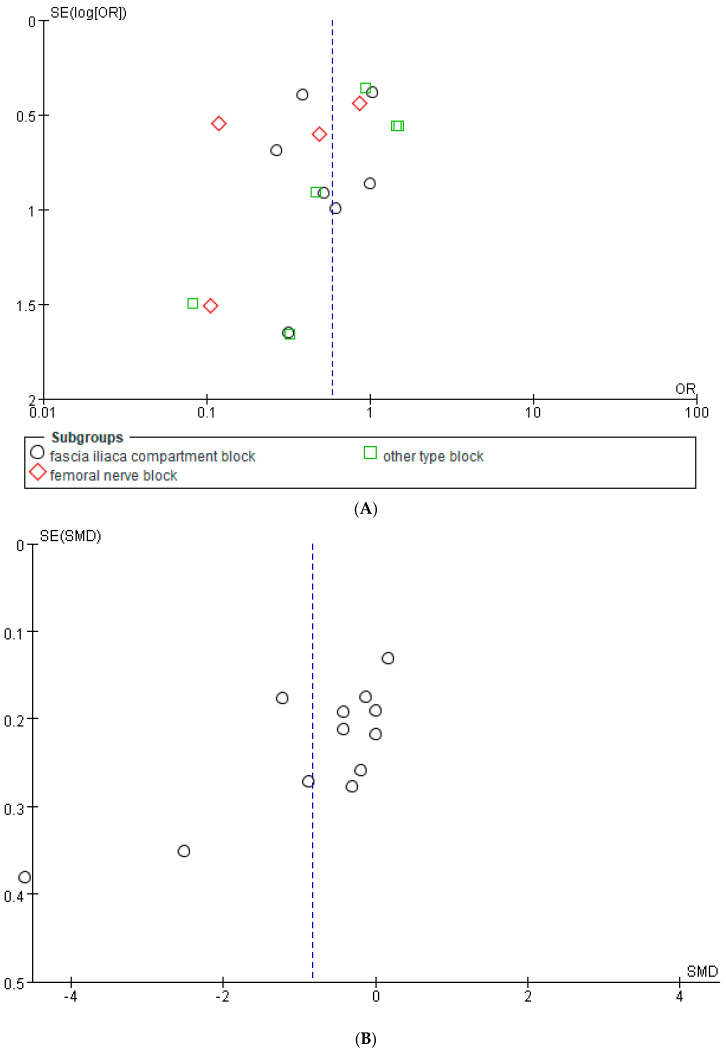
Funnel plot for publication bias. (**A**) Postoperative delirium at postoperative day three. (**B**) Postoperative pain scores. SE, standard error; OR, odds ratio.

Funnel plots and Egger’s tests were performed for POD (*p* = 0.1378) and postoperative pain scores (*p* = 0.2153) at postoperative day three, and no evidence of publication bias was identified (Figure 5). Studies regarding POD on postoperative day seven were not tested for publication bias due to the small number of included studies.

## 4. Discussion

This systematic review and meta-analysis showed that PNB, especially the fascia iliaca compartment and femoral nerve blocks, lowered the incidence of POD on postoperative day three. In participants without preexisting cognitive impairment, PNB lowered the occurrence of POD. However, a comparison between the nerve block and no-block groups revealed no effect of PNB on POD on postoperative day seven. The severity of the postoperative pain was attenuated when PNBs were performed. This study is meaningful in that it included a relatively large number of studies compared to previous meta-analyses, and analyzed the effect of different types of peripheral nerve blocks on the incidence of postoperative delirium in elderly patients undergoing hip surgery.

According to our meta-analysis, the implementation of PNB had a lowering effect on POD on postoperative day three, and this effect was particularly pronounced when a fascia iliaca compartment block or a femoral nerve block were performed. These results are consistent with those of previous studies showing that fascia iliaca compartment blocks and femoral nerve blocks relieve pain in patients with hip fracture, given that pain is a crucial risk factor for POD [43,44]. According to Hilton’s law, pain from hip fractures is mainly carried by the obturator, sciatic, and femoral nerves [45]. The femoral nerve covers the anterolateral region [45], where the main incision is made during a hip surgery. Moreover, the anterior capsule, predominantly supplied by the femoral and obturator nerves, has a high density of nociceptors and mechanoreceptors, making it a major source of pain in the hip joint [46]. Therefore, it is speculated that the fascia iliaca compartment block and femoral nerve block, which mainly target the femoral nerve, have a remarkable effect in reducing POD after a hip surgery.

In our study, a peripheral nerve block significantly reduced POD on postoperative day three, but not on postoperative day seven. Although these results may suggest that the PNB implementation only has a short-term impact on the occurrence of POD, it is difficult to draw firm conclusions from our meta-analysis alone because of the small number of studies that measured the incidence of delirium at postoperative day seven. In addition, among the studies included in the meta-analysis of POD on postoperative day seven, only one study performed a femoral nerve block, which reduced POD on postoperative day three, while two other studies performing a lumbar plexus block or lumbosacral plexus block showed no effect in reducing POD on postoperative day three. The impact of mid- to long-term cognitive impairment should be reexamined after further research.

In a previous study [47], PNB was effective in reducing POD in patients without underlying cognitive impairment. In this study, the performance of PNB did not differ regarding POD when patients with underlying cognitive impairment were included. However, in our study, PNB was effective in lowering the occurrence of POD, not only in the subgroup without baseline cognitive impairment, but also in general cases, including studies targeting patients with cognitive impairment. Conversely, when a subgroup analysis was conducted only on the studies that included patients with previous cognitive impairment, PNB showed no effects on POD occurrence. Nonetheless, because of the very small number of studies in this subgroup analysis, it is necessary to investigate more RCTs in the future to determine the effect of PNB on the incidence of POD in patients with cognitive impairment.

Li et al. [48] also examined the effects of nerve blocks on postoperative neurological changes via a meta-analysis. The authors of this meta-analysis placed no restrictions on the type of surgery or age of participants, and included not only peripheral nerve blocks, but also neuraxial blocks, such as spinal anesthesia or caudal blocks, which were excluded in our study. In this study, regional anesthesia did not reduce the incidence of POD in older adults or patients who underwent orthopedic surgery. In contrast, our study demonstrated that the POD incidence can be significantly reduced when PNB is performed in older patients undergoing hip surgery.

According to our meta-analysis, PNB significantly lowered the postoperative pain scores. Given that pain is a major risk factor for POD [12], this result correlates with the finding that POD was reduced in patients who underwent PNB. However, as heterogeneity was high in this analysis, caution should be exercised when interpreting the results.

Our study had several limitations. First, the method of measuring delirium occurrence was different in each included study. In many studies [18,31,36,37,38,40], delirium was measured using the Confusion Assessment Method (CAM) or MMSE scores; however, some studies used tools such as the Organic Brain Syndrome Scale [33], Delirium index [42], Delirium Rating Scale-R-98 [34], Short Portable Mental Status Questionnaire [29], and internal criteria of the study [28]; many studies did not record specific measurement methods [16,17,19,30,32,35,39,41]. Second, the timing of the PNB was different. Some studies [28,32,33,34] performed nerve blocks as soon as a femoral fracture was diagnosed or in the emergency room, and some studies [17,18,30,36,37,38,39,41,42] performed nerve blocks in the operating room before surgery. One study [19] conducted a nerve block immediately after skin closure, while another study [31] performed a nerve block twice by injecting a bolus shot and inserting a catheter. Third, the dose, volume, and concentration of local anesthetics used in peripheral nerve blocks were different between the studies. Even with the same type of PNB, the dose, volume, and concentration of injected local anesthetics were different. The dose and concentration of the local anesthetic affect the depth of the PNB [49], which can affect the degree of pain relief and ultimately, the development of postoperative delirium, where the pain is a risk factor. These different interventions may have affected the meta-analysis results. Fourth, reports on safety related to local anesthetics and PNB were insufficient in most studies. Local anesthetic systemic toxicity (LAST) is important, in particular, because it can be confused with POD, as it can cause central nervous system symptoms such as agitation or confusion [50]. Only one study [38] mentioned the absence of neurologic complications, while most of the other studies did not mention specific complications or investigated opioid-related complications, such as PONV. Lastly, some degree of heterogeneity was found in the subgroup analysis of the femoral nerve block effect on POD at postoperative day three and the meta-analysis of the pain score on postoperative day three.

In conclusion, perioperative PNB can reduce the incidence of POD in older adults undergoing hip fracture surgery. However, further investigation is required to verify these results. We believe that well-planned large clinical trials using standardized tools to assess POD, while reducing as many confounders as possible, may clarify the effect of perioperative PNB on cognitive status after a hip fracture surgery in older adults.

## Figures and Tables

**Table 1:** Study design and characteristics of included studies.

Source	Age (Number of Patients)		Type of Surgery	Type of Nerve Block	Measurement of Postoperative Delirium	Type of Local Anesthetics (Dose)	
	Control	Nerve Block				Bolus	Continuous Catheter
Gu 2021 [42]	73.4 ± 22.9 (45)	74.2 ± 20.7 (42)	femoral neck fracture, femoral intertrochanteric fracture, and femoral subtrochanteric fracture	Fascia iliaca compartment block, sacral plexus block, and superior cluneal nerve block	Delirium index	Fascia iliaca compartment block: 0.3% ropivacaine (30–35 mL) Sacral plexus block: 0.3% ropivacaine (5–10 mL) Superior cluneal nerve block: 0.3% ropivacaine (3–4 mL)	None
Hao 2019 [28]	72.5 ± 4.3 (42)	72.3 ± 3.8 (43)	Hip surgery (not specified)	Fascia iliaca compartment block	Internal criteria *	0.45% ropivacaine (30 mL)	None
Hua 2017 [16]	69.2 ± 4.0 (30)	68.7 ± 4.4 (30)	Hip replacement surgery	Fascia iliaca compartment block	NI	0.4% ropivacaine (35 mL)	None
Mouzopoulos 2009 [18]	73.1 ± 3.8 (105)	72.3 ± 4.1 (102)	Hemiarthroplasty, intramedullary nailing	Fascia iliaca compartment block	CAM	0.25% bupivacaine (0.3 mL/kg)	None
Loessin 2019 [17]	NI	NI	Hip surgery (not specified)	Fascia iliaca compartment block	NI	0.125% ropivacaine (40 mL)	0.2% ropivacaine (10 mL/h)
Nie 2015 [19]	68.2 ± 2.1 (53)	73.6 ± 2.1 (51)	Open reduction and internal fixation	Fascia iliaca compartment block	NI	0.5% ropivacaine (20 mL if BW < 50 kg, 25 mL if BW 50–70 kg, 30 mL if BW > 70 kg)	0.25% ropivacaine (0.1 mL/kg/h)
Wennberg 2019 [29]	84.9 ± 7.7 (60)	84.6 ± 6.7 (65)	Cervical, trochanteric, and subtrochanteric femur fracture surgery	Fascia iliaca compartment block	Short Portable Mental Status Questionnaire	0.2% ropivacaine (30 mL)	None
Yamamoto 2019 [30]	84.6 ± 7.8 (28)	84.7 ± 6.5 (25)	Internal fixation and bipolar hemiarthroplasty	Fascia iliaca compartment block	NI	0.25% levobupivacaine (40 mL)	None
Kullenberg 2004 [35]	82.7 ± 7.5 (40)	81.3 ± 6.5 (40)	Nail osteosynthesis and hemiendoplasty	Femoral nerve block	NI	0.75% ropivacaine (30 mL)	None
Morrison 2016 [31]	79.6 ± 27.4 (81)	81.1 ± 26.7 (72)	Femoral neck fracture, intertrochanteric fracture, and pericapsular fracture surgery	Femoral nerve block	CAM	0.5% bupivacaine (20 mL)	0.2% ropivacaine (15 mL bolus, then 5 mL/h)
Rowlands 2018 [32]	83.9 ± 6.2 (56)	83.0 ± 5.8 (55)	Hip surgery (not specified)	Femoral nerve block	NI	0.25% ropivacaine (0.5 mL/kg)	0.2% ropivacaine (5 mL/h)
Unneby 2020 [33]	84.4 ± 6.4 (120)	83.7 ± 7.1 (116)	Hip surgery (not specified)	Femoral nerve block	Organic Brain Syndrome Scale	0.25% ropivacaine (40 mL)	None
Uysal 2020 [34]	82.0 ± 6.8 (45)	81.4 ± 8.0 (46)	Trochanteric femur fracture surgery	Femoral nerve block	Delirium Rating Scale-R-98	0.25% bupivacaine (10 mL)	None
Bielka 2021 [39]	71.7 ± 2.2 (30) 73.0 ± 1.5 (30)	71 ± 3.7 (30)	Osteosynthesis of proximal femur	Psoas compartment block and sciatic nerve block	NI	Psoas compartment block: 0.5% bupivacaine (40 mL) Sciatic nerve block: 1.5% lidocaine (30 mL)	0.125% bupivacaine (6–8 mL/h)
Carvalho 2021 [40]	83.8 (29)	80.8 (28)	Hip prosthesis, dynamic screw, and femoral nail	Femoral and lateral cutaneous nerve block	CAM	Not specified	Not specified
Chen 2021 [41]	72.8 ± 5.9 (53)	73.9 ± 6.2 (53)	Lower limb internal fixation, total hip arthroplasty, replacement, and artificial femoral head replacement	Iliac fascial space block and sciatic nerve block	NI	0.5% ropivacaine (0.2 mL/kg)	None
Li 2018 [38]	68.3 ± 8.5 (30)	71.6 ± 7.2 (30)	Hip replacement surgery	Lumbar plexus nerve block	MMSE	0.375% ropivacaine (5 mL)	0.2% ropivacaine (basal 4 mL/h, bolus 2 mL, lockout time 30 min)
Mei 2017 [36]	74 ± 7 (66)	75 ± 6 (66)	Total hip arthroplasty	Lumbosacral plexus block	CAM, MMSE	0.5% ropivacaine (15 mL)	None
Tang 2021 [37]	78.0 ± 6.5 (55)	76.6 ± 7.0 (55)	Osteosynthesis, artificial femoral head replacement, and total hip replacement	Lumbosacral plexus block	CAM	0.25% ropivacaine (20 mL each)	None

Age is presented as mean ± standard deviation. Age of control group in study by Bielka et al. [39] is presented separately, according to the type of anesthesia. The first row is the group under spinal anesthesia, and the second row is the group under general anesthesia. BW: body weight; NI: no information; CAM: Confusion Assessment Method; MMSE: Mini-Mental State Examination. * Internal criteria; 1. Acute onset of mental changes and fluctuating course, 2. Inattention, 3. Disorganized thinking, 4. Altered levels of consciousness, needs to meet all four criteria.

## Data Availability

No new data were created or analyzed in this study.

## Supplementary Materials

The following supporting information can be downloaded at: https://www.mdpi.com/article/10.3390/jcm12072459/s1, File S1: Search strategy for each database; File S2: GRADE.
