# Supplementary material for: The Effect of Peripheral Nerve Block on Postoperative Delirium in Older Adults Undergoing Hip Surgery: A Systematic Review and Meta-Analysis of Randomized Controlled Trials
Source: J Clin Med. 2023 Mar 23;12(7):2459. doi: 10.3390/jcm12072459 (PMC10095174; doi:10.3390/jcm12072459)
Supplement: Supplementary file 1 [file jcm-12-02459-s001.zip › File S1.pdf]

File S1. Search strategy for each database

PubMed/MEDLINE

#1. nerve block OR block* OR nerve analges* OR analges*

#2. deliri* OR cognitive disorder OR cognitive OR cognitive impairment OR neurocognitive OR mental

#3. fractures, hip [MeSH Terms] OR trochanteric fractures [MeSH Terms] OR fractures, trochanteric [MeSH Terms] OR intertrochanteric fractures [MeSH Terms] OR fractures, intertrochanteric [MeSH Terms] OR subtrochanteric fractures [MeSH Terms] OR fractures, subtrochanteric

#4. hip OR trochanter* OR intertrochanter* OR subtrochanter* OR pertrochanter* OR femoral

#5. fracture OR surger* OR op*

#6. #4 AND #5

#7. #3 OR #6

#8. randomized controlled trial [pt]

#9. controlled clinical trial [pt]

#10. randomized [tiab] OR randomized [tiab]

#11. placebo [tiab]

#12. randomly [tiab]

#13. trial [tiab]

#14. groups [tiab]

#15. #8 OR #9 OR #10 OR #11 OR #12 OR #13 OR #14

#16. animals [mh] NOT humans [mh]

#17. #15 NOT #16

#18. #1 AND #2 AND #7 AND #17

Embase

#1. ‘nerve block’/exp OR ‘nerve block’ OR ‘nerve analgesia’/exp OR ‘nerve analgesia’

#2. ‘delirium’/exp OR ‘delirium’ OR cognitive* OR neurocognitive* OR mental

#3. ‘hip fracture’/exp OR ‘hip fracture’

#4. trochanter* OR hip OR intertrochanter* OR subtrochanter* OR pertrochanter* OR femoral

#5. fracture/exp OR fracture OR surgery/exp OR surger* OR operation/exp OR op*

#6. #4 AND #5

#7. #3 OR #6

#8. Random*:ab,ti OR ((clinical NEXT/1 trial*):de,ab,ti) OR placebo*:de,ab,ti OR ((double NEXT/1 blind*):ab,ti) OR group*

#9. #1 AND #2 AND #7 AND #8

The Cochrane Library

#1. (“nerve block” OR block* OR “nerve analges*” OR analges*):ti,ab,kw (Word variations have been searched)

#2. (deliri* OR cognitive* OR mental OR neurocognitive*):ti,ab,kw (Word variations have been searched)

#3. MeSH descriptor: [Fractures, Hip] explode all trees

#4. (hip OR trochanter* OR intertrochanter* OR subtrochanter* OR pertrochanter* OR femoral):ti,ab,kw (Word variations have been searched)

#5. (fracture OR surger* OR op*):ti,ab,kw (Word variations have been searched)

#6. #4 AND #5

#7. #3 OR #6

#8. #1 AND #2 AND #7 in Trials

Web of Science

#1. TS=(“nerve block”)

#2. TS=(deliri* OR “cognitive disorder” OR cognitive OR “cognitive impairment” OR neurocognitive OR mental)

#3. TS=(hip OR trochanter* OR intertrochanter* OR subtrochanter* OR pertrochanter* OR femoral)

#4. TS=(fracture OR surger* OR oper*)

#5. #3 AND #4

#6. TS=(random* OR “clinical NEXT/1 trial*” OR placebo* OR “double NEXT/1 blind*” OR group*)

#7. #1 AND #2 AND #5 AND #6
