# Supplementary material for: The Effect of Peripheral Nerve Block on Postoperative Delirium in Older Adults Undergoing Hip Surgery: A Systematic Review and Meta-Analysis of Randomized Controlled Trials
Source: J Clin Med. 2023 Mar 23;12(7):2459. doi: 10.3390/jcm12072459 (PMC10095174; doi:10.3390/jcm12072459)
Supplement: Supplementary file 1 [file jcm-12-02459-s001.zip › File S2, GRADE.pdf]

Certainty assessment							№ of patients		Effect		Certainty	Importance
№ of studies	Study design	Risk of bias	Inconsistency	Indirectness	Imprecision	Other considerations	PNB	control	Relative (95% CI)	Absolute (95% CI)		
**Postoperative pain**												
13	randomised trials	serious	not serious	not serious	not serious	none	721	741	-	SMD **0.83 lower** (1.36 lower to 0.3 lower)	⊕⊕⊕◯ Moderate	
**Postoperative delirium at postoperative day 3**												
17	randomised trials	not serious	not serious	not serious	not serious	none	111/793 (14.0%)	168/808 (20.8%)	**OR 0.59** (0.40 to 0.87)	**74 fewer per 1,000** (from 113 fewer to 22 fewer)	⊕⊕⊕⊕ High	
**Postoperative delirium at postoperative day 7**												
3	randomised trials	serious	not serious	not serious	not serious	none	97/201 (48.3%)	93/205 (45.4%)	**OR 1.26** (0.76 to 2.09)	**58 more per 1,000** (from 67 fewer to 181 more)	⊕⊕⊕◯ Moderate	

CI: confidence interval; **OR:** odds ratio; **SMD:** standardised mean difference
